# Exploring the Wisdom Structure: Validation of the Spanish New Short Three-Dimensional Wisdom Scale (3D-WS) and Its Explanatory Power on Psychological Health-Related Variables

**DOI:** 10.3389/fpsyg.2018.00692

**Published:** 2018-05-14

**Authors:** Javier García-Campayo, Yolanda L. del Hoyo, Alberto Barceló-Soler, Mayte Navarro-Gil, Luis Borao, Veronica Giarin, R. Raziel Tovar-Garcia, Jesus Montero-Marin

**Affiliations:** ^1^Red de Investigación en Actividades Preventivas y Promoción de la Salud, Zaragoza, Spain; ^2^Hospital Universitario Miguel Servet, Zaragoza, Spain; ^3^Department of Psychology and Sociology, University of Zaragoza, Zaragoza, Spain; ^4^Instituto de Investigación Sanitaria Aragón (IIS Aragón), Zaragoza, Spain; ^5^Unidad de Investigación de Atención Primaria, Zaragoza, Spain; ^6^Centro de Investigación y Desarrollo en Ciencias de la Salud, Universidad Autónoma de Nuevo León, San Nicolás de los Garza, Mexico

**Keywords:** wisdom, 3D-WS, validation, EFA, CFA, psychometrics, well-being, bifactor

## Abstract

**Introduction:** Personal wisdom has demonstrated important implications for the health of individuals. The aim of the present study was to validate a Spanish version of the Three-Dimensional Wisdom Scale (3D-WS), exploring the structure of a possible general factor, and assessing its explanatory power on psychological health-related variables.

**Methods:** A cross-sectional study design was used, with a total sample of 624 Spanish participants recruited on the Internet and randomly split into two halves. The following instruments were applied: 3D-WS, Purpose in Life (PIL), Multidimensional State Boredom Scale (MSBS), Positive and Negative Affect Scale (PANAS), and Difficulties in Emotion Regulation Scale (DERS). Factorial structures were analyzed through exploratory and confirmatory factor analysis (EFA and CFA), and the general factor was characterized by using bifactor models. The explanatory power of the 3D-WS was established by multiple regression.

**Results:** The original long and short versions of the 3D-WS were not replicated in the first subsample using EFA, and there was a high rate of cross-loadings. Thus, a new short 3D-WS was proposed by ordering the original items according to factorial weights. This three-correlated-factor (reflective, cognitive, and affective) proposal was tested by means of CFA in the second subsample, with adequate psychometrics and invariance, and a good fit (χ^2^/df = 1.98; CFI = 0.946; RMSEA = 0.056; 90% CI = 0.040-0.072). A bifactor structure, in which the reflective trait of wisdom was integrated into a general factor (G-Reflective) improved the model fit (χ^2^/df = 1.85; CFI = 0.959; RMSEA = 0.052; 90% CI = 0.035-0.070). The explained common variance of G-Reflective was 0.53; therefore, the new short 3D-WS should not be considered essentially unidimensional. The new short 3D-WS showed positive relationships with the PIL and PANAS-positive, and negative associations with the MSBS, PANAS-negative and DERS, contributing to explain all the referred variables. These results were consistent across subsamples.

**Conclusion:** The new short 3D-WS appears to be a reliable instrument for measuring wisdom in the Spanish general population. The reflective facet might influence the cognitive and affective wisdom components through the G-Reflective general factor. There seems to be a high explanatory power of the 3D-WS on psychological health-related variables. This study will facilitate the development of future research and psychological knowledge regarding wisdom.

## Introduction

Personal wisdom has increasingly become a research subject in psychology in previous years, despite the intrinsic difficulty of establishing a broadly accepted definition on what appears to be a very slippery subject. This may be a result of the inherently cultural nature attributed to wisdom (Takahashi and Overton, 2005). In general, it has recently been indicated that wisdom may have significant implications for individuals and health care systems via improvements in physical and mental health (Ardelt, 2000, 2003; Jeste et al., 2013; Webster et al., 2014), and quality of life (Ardelt, 1997, 2000; Jeste and Oswald, 2014), as well as psychological health-related outcomes, such as resilience (Jeste et al., 2013), happiness (Etezadi and Pushkar, 2013; Zacher et al., 2013), self-efficacy (Glück et al., 2013), life satisfaction (Ferrari et al., 2011; Le, 2011) and forgiveness (Taylor et al., 2011). In addition, wisdom may be beneficial for other individuals and society at large by promoting the well-being of other individuals, and improving the quality of social relationships (Ardelt, 1997, 2000; Jeste and Oswald, 2014). However, wisdom is a complex psychological construct -with various not necessarily equivalent operationalizations that are focused on different definitions to some extent (Glück et al., 2013)- that is extremely difficult to study.

There is a consensus from different theoretical orientations that wisdom is a multifaceted or multidimensional psychological concept, and some of its component facets or dimensions may feed and reinforce each other (Webster, 2003). Although not free of debate regarding what are the essential components of wisdom, both necessary and sufficient, and what constitutes predictors and consequences, one basic definition of wisdom that spans an especially broad range of facets of wisdom consider it formed by cognitive (general), reflective (self-related) and affective (other-related) components (Ardelt, 2011). This definition is able to generate a parsimonious concept compatible with modern and ancient descriptions of the topic (Clayton and Birren, 1980; Ardelt, 2003). The cognitive component includes the ability to understand and comprehend the deeper meaning of life events, including the ambiguity of human nature, the limits of knowledge and the uncertainty of life (Ardelt, 2000, 2003). The reflective dimension, which seems to be essential to facilitate referred understanding and cognitions, consists of the ability to acquire different perspectives, overcome self-centeredness, subjectivity and projections, attain insights into the true nature of things and motivations, and avoid blaming other individuals for one’s own circumstances (Ardelt, 2003). The affective factor is based on the presence of positive emotions and a sympathetic and compassionate behavior toward other individuals, as well as the absence of indifferent or negative feelings and behaviors toward other individuals. It may also depend on the reflective dimension because a deep understanding of life and individuals from a positive point of view is only possible if one can perceive reality as it is with no major biases (Ardelt, 2003). This model considers wisdom as an integration of the three above-mentioned facets, which are conceptualized as developmental personality qualities that may be measured by the long and short versions of the “Three-Dimensional Wisdom Scale” (3D-WS) (Ardelt, 2003; Thomas et al., 2017). There have been shown to be inter-factorial correlations between the 3D-WS factors in the original study with regard to ‘reflective–cognitive’ of 0.41-0.50, ‘reflective–affective’ 0.46-0.50, and ‘affective–cognitive’ 0.30-0.33. The factor loadings from a possible general second order factor were of 0.83-0.84 for the reflective, 0.59-0.61 for the affective, and 0.50-0.52 for the cognitive. According to the original author, all of this suggests the reflective facet might be fostering both cognitive and affective characteristics of wisdom (Ardelt, 2003). The 3D-WS was originally developed in English. To date, no version exists in Spanish that enables investigations of the implications of this wisdom model in Spanish-speaking countries.

In this context, the main aim of the present study was to validate a new Spanish-language version of the 3D-WS. As secondary objectives, we aimed to explore the structure of a possible general factor and the influence of the reflective characteristics on all the aspects of wisdom; as well as to estimate the explanatory power of the 3D-WS on psychological health-related variables to evaluate the extent to which wisdom may contribute to well-being. To date, no Spanish-language studies have evaluated potential relationships between wisdom and psychological outcomes, such as purpose in life, boredom, positive and negative affectivity, and emotional regulation. Purpose in life has been of interest in existential psychotherapy (Crumbaugh and Maholick, 1969), and it has shown negative associations with depression and positive relationships with psychological well-being (Bonebright et al., 2000). Boredom has been associated with several psychiatric disorders, such as anxiety, depression, somatisation, overeating and binge eating, pathological gambling, and substance abuse (Alda et al., 2015). In general, positive and negative affectivity explain an important portion of psychological well-being (Menk Otto et al., 2010). Emotion regulation is a central component of mental health, and its imbalances may underlie several mental disorders (Mennin and Farach, 2007). Therefore, we started with the exploratory assumption that a new Spanish version of the 3D-WS could be validated with adequate psychometric properties. We also stated that the reflective facet might be contributing to the general factor to a greater extent than the other components. Finally, we hypothesized that wisdom may significantly explain all of the above-mentioned psychological outcomes, and it may be positively related to purpose in life and positive affectivity, and negatively to boredom, negative affectivity and the absence of emotional regulation.

## Materials and Methods

### Design

An analytical cross-sectional design was developed within a validation study, using back-translations of the original questionnaire and an online survey.

### Participants, Data Collection and Ethics

The online survey was developed on a commercial system^1^, and it was disseminated through several websites from the authors’ scientific research webpage. Individuals were invited to participate in research on “general aspects related to wisdom.” The link to the survey was accessible from September 2016 to June 2017. Overall, 1,808 participants accessed the link, and 1,737 individuals voluntarily agreed to participate. Participants who did not complete all items of the 3D-WS validation questionnaire (*n* = 937) were excluded. Those participants with nationalities or provenances other than Spain (*n* = 176) were also excluded, given the intention that everyone should use a similar standard variety of Spanish and be able to interpret the questionnaire statements in the same way. Therefore, 624 Spanish individuals were recruited. The majority of the participants were female (75.6%), with a mean age of 44.70 (*SD* = 12.61; Range = 18-75), and mainly with a partner in a stable relationship (63.1%), a university education (79.5%) and in employment (72.9%). The total sample was randomly split into two halves (312 participants each) in order to develop exploratory and confirmatory analyses using different subsamples. A sample size of *n* = 312 subjects, with a null hypothesis that RMSEA would be equal to or less than 0.050 if the true value was 0.080 (close fit) and an alpha equal to 0.05 level, produces power coefficients ranging from 0.72 (lower-powered analysis: exploratory factor analysis of the short 3D-WS using a bifactor model, with 33 degrees of freedom) to 0.99 (higher-powered analysis: exploratory factor analysis of the long 3D-WS, with 627 degrees of freedom) (MacCallum et al., 1996). The protocol used in this study was approved by the Ethical Committee of the regional health authority of Aragon (CEICA, PI16/0117), and all participants submitted a written informed consent form online attesting to their willingness to participate.

### Validation Procedure

We initially obtained permission from the original author (Ardelt, 2003) to translate into Spanish and validate the 3D-WS. Two researchers who were aware of the questionnaire’s objectives subsequently performed the initial translation from English to Spanish. Each researcher translated the questionnaire separately. Two bilingual linguistic experts, who had no specific knowledge regarding the instrument, produced back-translations. A native English-speaking teacher subsequently determined whether the two English versions were equivalent, and differences between the translations were solved through mutual agreement. An accepted guideline for cross-cultural adaptations was followed (Guillemin et al., 1993). The final Spanish version of the 3D-WS is shown in Additional File 1, and its corresponding English version in Additional File 2.

### Measures

#### Socio-Demographic

The general socio-demographic information obtained from the participants included age, sex, nationality (Spain, South America, Central America, others), marital status (with partner, single, divorced, or widower), level of education (primary, secondary, or university) and employment situation (student, employed, on sick leave, unemployed).

#### Three-Dimensional Wisdom Scale (3D-WS)

The long 3D-WS (Ardelt, 2003) includes 39 items: 14 items for the cognitive dimension (e.g., “I am hesitant about making important decisions after thinking about them”), 12 items for the reflective dimension (e.g., “When I look back on what has happened to me, I can’t help feeling resentful”), and 13 items for the affective dimension (e.g., “I don’t like to get involved in listening to another person’s troubles”). The short 3D-WS (Thomas et al., 2017) includes only 12 items of the total pool, with four items for each of the three dimensions previously described. The items are self-rated using five options, and they are scaled from 1 (strongly agree or definitely true of myself) to 5 (strongly disagree or not true of myself); 5 items from the reflective dimension and 3 items from the affective dimension are reverse-scored (they are marked with an “*r*” in **Table 2**). The scale structure also supports a total second-order factor of wisdom, in which higher scores indicate greater wisdom levels, with adequate psychometric properties in its first proposal and both the long and short versions (Ardelt, 2003; Thomas et al., 2017).

#### Purpose in Life (PIL)

The PIL (Crumbaugh and Maholick, 1969) is one of the tools most commonly employed to measure the meaning of life. It was used in Part A of the questionnaire, which has 20 items Likert-type distributed among the components of general perception of the meaning of life (e.g., “My personal existence is: utterly meaningless, without purpose/purposeful and meaningful”) and satisfaction with life (e.g., “Life to me seems: completely routine/always exciting”), with a general total score of purpose in life, in which higher scores indicate a higher purpose in life level. As indicated, each item has specific response anchors with respect to categories 1–7, whereas category 4 entails a neutral attitude toward the statements. The Spanish version of the PIL has shown good psychometric properties (Martínez et al., 2012), with a total alpha value in the present study for the total scale of α = 0.94 and a 95% confidence interval (95% CI) = 0.93-0.95, using Fisher’s method (Fisher, 1950) because of its efficiency (Dominguez-Lara and Merino-Soto, 2015) [general perception of the meaning of life α = 0.90 (95% CI = 0.88-0.91), and satisfaction with life α = 0.79 (95% CI = 0.76-0.82)].

#### Multidimensional State Boredom Scale (MSBS)

The MSBS (Fallman et al., 2013) is a self-reported 29-item questionnaire that measures state boredom using the dimensions of disengagement (e.g., “I am wasting time that would be better spent on something else”), high arousal (e.g., “Everything seems to be irritating me right now”), low arousal (e.g., “It seems like there’s no one around for me to talk to”), inattention (e.g., “I am easily distracted”), and time perception (e.g., “Time is passing by slower than usual”). It also permits a total score, in which higher scores indicate higher boredom levels. Each item is rated on a scale from 1 (strongly disagree) to 7 (strongly agree) in relation to the respondent’s present experience. The scale has recently been validated in Spanish with appropriate psychometric parameters (Alda et al., 2015), with an alpha value in the present study for the total scale of α = 0.97 (95% CI = 0.97-0.98) [disengagement α = 0.94 (95% CI = 0.93-0.95); high arousal α = 0.87 (95% CI = 0.85-0.89), low arousal α = 0.92 (95% CI = 0.90-0.93), inattention α = 0.91 (95% CI = 0.89-0.92), time perception α = 0.92 (95% CI = 0.91-0.93)].

#### Positive and Negative Affect Scale (PANAS)

The PANAS is a brief measure of positive (e.g., “Enthusiastic”), and negative (e.g., “Distressed”) affectivity (Watson et al., 1988). It consists of a list of 20 adjectives, 10 per subscale, rated on a 5-point Likert-type scale from 1 (very slightly or not at all) to 5 (extremely). Present moment temporary instructions were used in this study. Higher scores indicate greater levels of positive/negative affectivity. This questionnaire has been validated in Spanish with good psychometrics (Sandín et al., 1999), with α = 0.92 (95% CI = 0.91-0.93) and α = 0.91 (95% CI = 0.89-0.92) for the positive and negative scales, respectively, in the present study.

#### Difficulties in Emotion Regulation Scale (DERS)

The DERS is a questionnaire that assesses aspects of the emotion regulation process in which individuals may have difficulties. The Spanish version (Hervás and Jódar, 2008) consists of 28 items grouped into the subscales of lack of emotional awareness (e.g., “I am attentive to my feelings” -item reversed), lack of emotional clarity (e.g., “I have difficulty making sense out of my feelings”), non-acceptance (e.g., “When I’m upset, I become angry with myself for feeling that way”), goals (e.g., “When I’m upset, I have difficulty concentrating”), and impulse (e.g., “When I’m upset, I have difficulty controlling my behaviors”); it also permits a global score. Participants are asked to indicate how often the items apply to themselves, with responses that range from 1 (almost never) to 5 (almost always). Higher scores indicate greater difficulties in emotion regulation. This scale has shown evidence of adequate psychometric properties (Hervás and Jódar, 2008), with an internal consistence in the present study for the total scale of α = 0.96 (95% CI = 0.95-0.97) [lack of emotional awareness α = 0.86 (95% CI = 0.84-0.87); lack of emotional clarity α = 0.83 (95% CI = 0.80-0.85); non-acceptance α = 0.94 (95% CI = 0.93-0.95); goals α = 0.91 (95% CI = 0.89-0.92), impulse α = 0.93 (95% CI = 0.92-0.94)].

### Statistical Analysis

The socio-demographics were described using means (SDs) and frequencies (percentages) according to their nature, and possible differences between subsamples were tested using the *t* for independent groups and χ^2^ (or Fisher when necessary) tests. The items behavior was assessed using means (SDs), skewness, kurtosis and item-rest (factor/total) correlations. Mardia’s coefficients (Mardia, 1974) were calculated to evaluate their multivariate distribution. We verified the KMO sampling adequacy values, the Barlett’s test of sphericity on the redundancy levels and the matrix determinants to discard multi-collinearity problems (Muthén and Kaplan, 1992; Field, 2000).

Exploratory factor analysis (EFA) -using subsample 1- was conducted to discover the underlying factorial structure of the 3D-WS items. Schwartz’s Bayesian Information Criterion (BIC) was used as a dimensionality test to decide the number of factors to be retained. The unweighted least squares (ULS) method, with correcting for robust mean and variance-scaled, was employed for factor extraction in view of its robustness (Jöreskog, 1977). ULS does not provide significance p-values for the parameters; however: (a) it does not require distributional assumptions; (b) it is robust and typically converges because of its high efficiency in terms of computation; and (c) it tends to supply less biased estimates of the true parameter values than classical methods or far more complex procedures (Knol and Berger, 1991; Parry and McArdle, 1991; Briggs and MacCallum, 2003; Lee et al., 2012). Polychoric correlations, which are specially adapted to the analysis of relationships between polytomous categorical variables, were used to build the input matrices. The raw loading matrices were rotated using the Promin procedure, which allows factors to be oblique so that factor simplicity is maximized, without the assumption that all the variables are pure measures of a single dimension (Lorenzo-Seva, 1999; Ferrando and Lorenzo-Seva, 2000). Uniqueness terms (δ) were calculated as a measure of item unexplained variance. We evaluated factorial simplicity by means of: (a) the index of factor simplicity (IFS); (b) the scale fit index (SFI); (c) Bentler’s scale-free matrix measure; and d) hyperplane counts. IFS and SFI values of 0.80 are meritorious; Bentler’s measure ranges from 0 for very complex structures, to 1 for very simple ones; and hyperplane counts (loadings essentially zero except for random error) were estimated through the -0.15/+0.15 interval and using the Kaiser and Cerny procedure (Fleming, 2003). Factor scores were calculated by means of Bayes Expected a Posteriori (EAP) estimates because these scores have the highest correlations with the common factors they measure (Mulaik, 2010). Effectiveness and quality of factor score estimates were quantified by using the factor determinacy index (FDI) and marginal reliability estimates. FDI is the correlation between the factor score estimates and the levels on the latent factors they estimate (Beauducel, 2011), and values of around 0.80 are adequate (Gorsuch, 1983). Marginal reliability was obtained by FDI squared, and it is interpreted as the reliability of the corresponding factor score estimates (Brown and Croudace, 2015). Construct replicability, the proportion of the factor variance that can be accounted for by its indicators, was measured by the H index -bounded between 0 and 1, with reasonable values when ≥0.70 (Hancock and Mueller, 2000), or more strictly ≥0.80 (Rodriguez et al., 2016). We explored closeness to unidimensionality by the mean of item residual absolute loadings (MIREAL) and the explained common variance (ECV). MIREAL is a measure of departure from unidimensionality, with <0.30 indicating no substantial bias if a unidimensional solution is fitted (Grice, 2001; Ferrando and Loranzo-Seva, 2017). ECV represents the proportion of common variance attributable to the general factor, and it should be in the range of 0.70-0.85 if a solution is to be accepted as unidimensional (Rodriguez et al., 2016). We tested an exploratory second order factor solution (Schmid and Leiman, 1957) and an exploratory bifactor model as two general factor (G) approaches for wisdom that would reflect what is common to all the items.

Confirmatory factor analysis (CFA) -using subsample 2- was used to ensure a clear distinction between the factors emerged from the EFA by loading each item onto its corresponding single component, all of them correlated. The Maximum Likelihood method (ML) was used, which employs Pearson correlations based on the covariance matrix as input data. The ML method is the most popular structural equation modeling (SEM) estimation procedure, as it provides asymptotically unbiased and consistent parameter estimates (Bollen, 1989), and permits inferential estimations based on the χ^2^ distribution, providing significance *p*-values. It implies the assumption of multivariate normality -particularly in terms of skewness (Coenders and Saris, 1995), but it is relatively robust to its non-observance (Hu and Bentler, 1999; Schermelleh-Engel et al., 2003). This method also assumes the continuous measurement of both latent and observed variables (DiStefano, 2002); however, the covariance matrix enables robust analysis to be made of ordinal data when the latent variables present more than one indicator (Coenders et al., 1997). From an analytical perspective, inter-factor correlations, standardized factor saturations (λ), uniqueness terms and discrepancy values -as unstandardized residual covariance estimates- were taken into account. From a general perspective, the goodness-of-fit was assessed by chi-square (χ^2^), chi-square/degrees of freedom (χ^2^/df), the comparative fit index (CFI) and the root mean square error of approximation (RMSEA). χ^2^ is very sensitive to sample size (Bollen and Long, 1993), so use was made of χ^2^/df, which indicates a good fit when <5, and an excellent fit if <3 (Hu and Bentler, 1999; Schermelleh-Engel et al., 2003). CFI examines the discrepancy between the data and the hypothesized model while adjusting for the sample size, and it indicates adequate fit with a value of >0.90 and an excellent fit >0.95 (Burnham and Anderson, 1998; Hu and Bentler, 1999). RMSEA is a measurement of the error of approximation to the population, and it indicates adequate fit when <0.08 and an excellent fit <0.06 (Burnham and Anderson, 1998; Hu and Bentler, 1999). An estimation was also made of the average variance extracted (AVE), as the amount of variance that is captured by the construct in relation to the variance due to measurement error. It shows good values of construct convergent validity when ≥0.50, but also has acceptable values if it is around 0.40 and composite reliability (CR) is >0.60. In addition, when AVE values are greater than the squared correlation between factors, it can be said there are discriminant validity among them (Fornell and Larcker, 1981).

**FIGURE 1:**
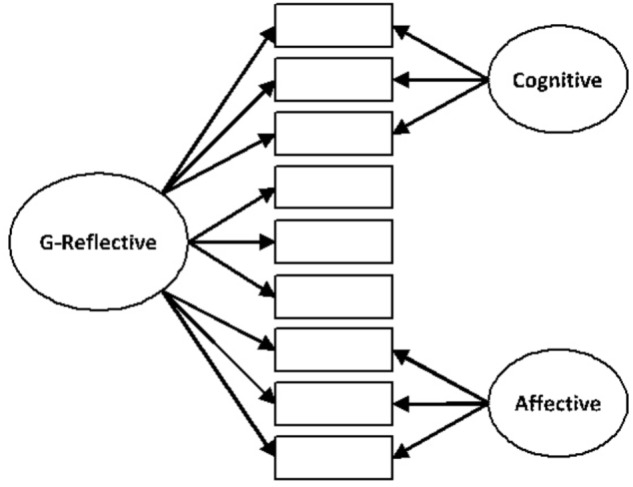
Hypothetical G-reflective structure of wisdom by means of the G-Reflective bifactor model without considering the final number of items.

The structure of the possible general factor of the new proposed 3D-WS was evaluated -using CFA and subsample 2- by testing a second-order solution, as well as a bifactor model that would reflect the influence of the reflective characteristics on the other wisdom aspects through the G-Reflective general factor (**Figure 1**). The omega CR for the total scale (ω) and for each subscale (ω_S_) were calculated, which may be interpreted as the square of the correlation between the scale (ω) -or subscale (ω_S_)- score and the latent variable common to the corresponding indicators. This reliability value has the advantage of taking into account the strength of association between items and constructs as well as item-specific measurement errors, while providing a more realistic estimate of true reliability than other classical methods (McDonald, 1999). We also estimated the omega hierarchical (ω_H_), as the proportion of reliable variance in total scores that can be attributed to the single general factor, and the omega hierarchical subscale (ω_HS_), as the proportion of reliable variance associated with each factor once partitioning out variance associated with the single general factor (Reise, 2012; Green and Yang, 2015). The percentage of uncontaminated correlations (PUC) were also estimated, as the number of correlations between items from different factors divided by the total number of correlations, which indicates the proportion of correlations reflecting the general factor. When ECV and PUC are >0.70 common variance can be regarded as essentially unidimensional (Rodriguez et al., 2016).

The configurational, metric, scalar and strict invariance of the new 3D-WS model across subsamples, and age, sex and level of studies -as relevant socio-demographic factors that might affect wisdom (Ardelt, 2003) and that are recommended in validation studies (Ayman and Korabik, 2010)- was sequentially evaluated using the ML method (Van de Schoot et al., 2012). A nested model adding covariances between latent factors was also incorporated to the strict invariance model. These five nested models were compared in order to allow some degree of invariance. Owing to the sensitivity to sample size of changes in χ^2^ (Hair et al., 1999), we ensured that both decreases in CFI and increases in RMSEA were ≤0.010 and ≤0.015, respectively (Chen, 2007). Because the goodness-of-fit indices corrected for parsimony (e.g., RMSEA) can be improved with the addition of model constraints, they were considered to be random. Possible differences in latent factor means (ΔMn) were tested across subsample, age, sex and level of studies using structured means modelling (SMM), and by setting the means of ‘subsample 1,’ ‘<55 years,’ ‘males,’ and ‘primary/secondary education’ to 0 (Sörbom, 1974). Effect sizes (*ES*s) of differences in latent means were also assessed by using Cohen’s *d*, dividing the referred differences by the within-groups pooled variance estimate for scores on latent variables (Hancock, 2001).

The relationships between the EAP estimates of the long 3D-WS and the new proposed 3D-WS were assessed across subsamples by applying Pearson’s *r* coefficients, adjusting for correlated errors (adj-*r*) (Levy, 1967). The raw correlations between the new proposed 3D-WS and the psychological health-related variables were estimated by applying *r* coefficients, and the explanatory power of the new proposed 3D-WS factors in relation to the psychological health-related variables by multiple linear regression models, which were examined using analysis of variance. The total scores of the psychological health-related variables were considered dependent variables, whereas the new proposed 3D-WS factors by EPA estimates were considered independent variables. Adjusted multiple determination coefficients (*R*^2^) were calculated to evaluate the explanatory power of the new proposed 3D-WS. The individual contribution of the independent variables in each regression model was estimated via calculation of the standardized slope coefficients (Beta). The Wald test was used to evaluate the significance of the contribution of each independent variable (Etxeberrìa, 2007). The assumptions of regression were tested using the *K*-*S* test over the conditional distribution of residuals, in order to check whether they were normally distributed; the Durbin-Watson test, in order to rule out possible autocorrelations in the error terms; as well as tolerance (T) and variance inflation factor (VIF) values, in order to discard collinearity problems (Martínez-González et al., 2006).

All the tests were bilateral and were performed with a significance level of α < 0.05. Data analysis was conducted with the SPSS-19, FACTOR-10, SIMLOAD, and AMOS-7 statistical packages.

## Results

### Socio-Demographics

The socio-demographics of the study participants, depending on their randomly selected subsample, are shown in **Table 1**. No significant differences were found between them in terms of age, sex, marital status, education level, or employment status.

**Table 1:** Socio-demographics of the participants according to subsample.

Variables/subsamples		Subsample 1 (*n* = 312)		Subsample 2 (*n* = 312)		
		*Mn*	*SD*	*Mn*	*SD*	*p*
Age		44.51	11.50	44.88	10.53	0.678
				
		**Freq.**	**%**	**Freq.**	**%**	

Sex	Female	240	76.9	232	74.4	0.454
	Male	72	23.1	80	25.6	
						
Marital status	With partner	205	65.7	189	60.6	0.411
	Single	65	20.8	69	22.1	
	Separated	35	11.2	48	15.4	
	Widower	7	2.2	6	1.9	
						
Education	Primary	15	4.8	15	4.8	0.803
	Secondary	46	14.8	52	16.7	
	University	251	80.4	245	78.5	
						
Employment	Student	30	9.6	22	7.1	0.630
	Employed	226	72.4	229	73.4	
	Sick leave	19	6.1	18	5.8	
	Unemployed	37	11.9	43	13.7	

*Mn, mean; SD, standard deviation; Freq., frequencies; %, percentages.*

### Exploratory Factor Analyses

#### Long 3D-WS

The descriptive statistics of all 3D-WS items (subsample 1) are shown in **Table 2**. All the items showed item-rest correlations in the same direction, with low values in some cases. Results of the BIC dimensionality test advised a 3-factor solution (**Table 3**). This solution explained 38.3% of the total variance, and only 20 out of 39 items (51.3%) loaded onto their theoretical belonging factor (**Table 4**). Goodness of model-data fit for the 3-factor proposal (**Table 5**), and general simplicity values were adequate (IFS = 0.89; SFI = 0.82; Bentler = 0.98). However, individual IFS values (**Table 4**), and the SFI value for the second factor (SFI_1_ = 0.89; SFI_2_ = 0.75; SFI_3_ = 0.81) suggested there was space for improvement. The factor determinacy of the EAP scores was good (FDI = 0.97 in all the factors). The marginal reliability estimates were appropriate (*F*_1_ = 0.93; *F*_2_ = 0.95; *F*_3_ = 0.93). Construct replicability was good (*H*_1_ = 0.91, *H*_2_ = 0.87, *H*_3_ = 0.88). The inter-factor correlations were moderately high (‘reflective-affective’ ϕ = 0.48, ‘reflective–cognitive’ ϕ = 0.54, ‘affective- cognitive’ ϕ = 0.46).

**Table 2:** Descriptive statistics of the Spanish 3D-WS items^∗^.

Factor	Item	*Mn*	*SD*	Skew	Kurt	Item-rest (*f*)	Item-rest (*t*)
Reflective	6	3.41	1.20	-0.36	-0.80	0.43	0.39
	10	3.70	1.03	-0.53	-0.32	0.38	0.51
	16r	4.16	0.79	-1.08	2.09	0.40	0.17
	18r	3.85	0.83	-0.51	0.00	0.45	0.26
	20r	4.10	0.78	-0.86	1.01	0.39	0.17
	23	3.96	1.06	-0.99	0.45	0.55	0.56
	26	3.62	1.06	-0.54	-0.35	0.62	0.49
	29	3.56	1.10	-0.70	-0.10	0.60	0.33
	32r	3.81	0.91	-0.76	0.43	0.37	0.20
	35r	3.77	0.90	-0.66	0.25	0.41	0.16
	37	3.95	1.03	-0.79	0.01	0.55	0.54
	39	3.90	0.93	-0.84	0.61	0.51	0.31

Affective	2	3.12	1.18	-0.06	-0.93	0.28	0.28
	4	3.85	1.18	-0.85	-0.12	0.21	0.31
	8	3.21	1.22	-0.12	-1.09	0.42	0.38
	12r	3.14	1.10	-0.17	-0.92	0.27	0.18
	14	4.21	0.88	-1.26	1.74	0.43	0.32
	17r	4.23	0.71	-0.53	-0.31	0.36	0.21
	19	3.82	1.03	-0.74	0.04	0.42	0.31
	21r	3.79	1.01	-0.78	0.33	0.40	0.28
	24	4.13	0.90	-1.09	1.21	0.33	0.35
	27	3.71	1.07	-0.66	-0.29	0.34	0.31
	30	3.46	1.01	-0.47	-0.12	0.39	0.41
	33	4.08	0.90	-0.82	0.08	0.37	0.29
	36	3.38	1.00	-0.38	-0.43	0.42	0.43

Cognitive	1	4.12	0.99	-1.15	0.93	0.20	0.18
	3	3.72	1.12	-0.79	-0.18	0.38	0.45
	5	3.89	1.00	-0.74	0.07	0.47	0.37
	7	4.42	0.81	-1.72	3.53	0.48	0.43
	9	3.90	1.04	-0.84	0.17	0.50	0.43
	11	3.32	1.04	-1.70	2.28	0.47	0.39
	13	3.70	1.01	-0.64	-0.20	0.41	0.39
	15	4.26	0.95	-1.45	1.82	0.54	0.42
	22	3.54	1.14	-0.55	-0.39	0.34	0.42
	25	3.33	1.01	-0.05	-0.57	0.17	0.27
	28	3.29	1.03	-0.36	-0.46	0.39	0.46
	31	3.50	1.04	-0.32	-0.57	0.22	0.17
	34	3.38	1.12	-0.38	-0.67	0.33	0.47
	38	3.66	1.12	-0.65	-0.42	0.17	0.11

*^∗^Subsample 1. Factor, theoretical belonging factor; Item, item number; Mn, mean; SD, standard deviation; skew, skewness; kurt, kurtosis; item-rest, discrimination coefficient for the factor (f) and total (t); r, reversed item.*

**Table 3:** 3D-WS dimensionality tests.

n° factors	BIC
Subsample 1	39 items 3D-WS original
0	18,661.78
1	2,144.29
2	1,967.87
3	1,866.25^∗^
4	1,895.80
5	1,984.63
Subsample 1	12 items 3D-WS original
0	1,990.04
1	265.34^∗^
2	267.78
3	314.57
Subsample 1	12 items 3D-WS proposed
0	2,545.99
1	403.24
2	335.16
3	323.12^∗^
4	372.66
5	433.70
Subsample 2	12 items 3D-WS proposed
0	2,595.14
1	469.80
2	403.09
3	326.69^∗^
4	377.30
5	430.16

*Results based on Schwartz’s Bayesian Information Criterion (BIC) dimensionality test. ^∗^Advised number of factors.*

**Table 4:** Exploratory factor analyses of the Spanish long 3D-WS^∗^.

		Long three-correlated-factors						Long exploratory bifactor					
Factor	item	λ_1_	λ_2_	λ_3_	S-L	δ	IFS^†^	λ_1_	λ_2_	λ_3_	G	δ	IFS^†^
Reflective	6	0.57	-0.17	0.09	0.39	0.68	0.85	0.26	-0.02	0.44	0.21	0.67	0.65
	10	0.31	-0.06	0.42	0.50	0.63	0.55	0.35	-0.14	0.19	0.46	0.63	0.55
	16r	0.03	0.69	-0.13	0.37	0.57	0.95	-0.10	0.63	0.01	0.37	0.50	0.96
	18r	-0.03	0.70	0.08	0.48	0.48	0.98	0.07	0.61	-0.06	0.47	0.38	0.97
	20r	-0.02	0.78	-0.14	0.38	0.48	0.96	-0.18	0.59	-0.03	0.46	0.48	0.87
	23	0.64	-0.10	0.17	0.54	0.51	0.87	0.19	-0.16	0.46	0.48	0.49	0.66
	26	0.81	0.06	-0.14	0.54	0.41	0.95	0.08	0.18	0.63	0.37	0.39	0.87
	29	0.81	0.10	-0.15	0.57	0.37	0.93	-0.03	0.06	0.62	0.51	0.36	0.98
	32r	0.04	0.66	-0.11	0.37	0.61	0.96	-0.19	0.42	0.01	0.47	0.61	0.76
	35r	0.06	0.58	-0.07	0.36	0.67	0.96	-0.05	0.51	0.03	0.35	0.63	0.98
	37	0.74	-0.12	0.09	0.55	0.45	0.94	0.22	-0.05	0.55	0.41	0.45	0.79
	39	0.42	0.40	-0.13	0.47	0.60	0.43	-0.05	0.35	0.31	0.43	0.60	0.49

Affective	2	0.20	-0.09	0.39	0.28	0.87	0.44	0.32	-0.08	-0.06	0.16	0.84	0.79
	4	-0.04	0.02	0.48	0.33	0.78	0.99	0.33	-0.12	-0.08	0.36	0.78	0.76
	8	0.30	-0.09	0.33	0.41	0.74	0.45	0.42	0.02	0.21	0.24	0.69	0.71
	12r	0.17	0.25	0.19	0.28	0.76	0.24	-0.05	0.32	0.15	0.24	0.82	0.71
	14	0.22	0.30	0.57	0.45	0.57	0.56	0.29	-0.01	-0.24	0.57	0.56	0.52
	17r	-0.08	0.48	0.16	0.36	0.71	0.83	0.02	0.26	-0.09	0.45	0.72	0.83
	19	0.26	-0.03	0.23	0.34	0.83	0.50	0.24	-0.03	0.18	0.28	0.82	0.56
	21r	0.04	0.38	0.22	0.44	0.70	0.64	0.20	0.31	0.00	0.40	0.66	0.61
	24	0.19	0.33	-0.11	0.42	0.83	0.56	0.12	-0.01	0.09	0.48	0.74	0.57
	27	0.03	0.28	0.23	0.37	0.79	0.53	-0.01	-0.04	-0.03	0.54	0.71	0.57
	30	0.51	0.14	-0.08	0.41	0.70	0.86	-0.11	-0.01	0.38	0.46	0.64	0.90
	33	-0.03	0.43	0.22	0.41	0.70	0.71	-0.06	0.04	-0.08	0.63	0.59	0.41
	36	0.50	0.21	-0.01	0.50	0.62	0.78	0.08	0.21	0.37	0.41	0.61	0.61

Cognitive	1	-0.04	-0.09	0.39	0.19	0.89	0.91	0.34	-0.08	-0.06	0.13	0.88	0.89
	3	0.38	-0.01	0.26	0.47	0.69	0.59	0.26	-0.05	0.26	0.41	0.69	0.47
	5	0.05	-0.09	0.59	0.41	0.66	0.96	0.57	-0.06	-0.01	0.29	0.62	0.98
	7	0.09	0.00	0.65	0.54	0.51	0.97	0.58	-0.03	0.01	0.44	0.48	0.99
	9	0.17	-0.02	0.47	0.45	0.68	0.84	0.36	-0.13	0.08	0.44	0.68	0.78
	11	0.03	0.24	0.48	0.52	0.59	0.72	0.28	-0.01	-0.04	0.59	0.58	0.96
	13	-0.11	-0.15	0.77	0.38	0.56	0.92	0.64	-0.18	-0.15	0.30	0.55	0.82
	15	-0.09	-0.03	0.77	0.47	0.49	0.98	0.61	-0.12	-0.14	0.43	0.48	0.88
	22	0.42	0.01	0.14	0.42	0.73	0.85	0.12	-0.09	0.29	0.42	0.72	0.70
	25	0.01	0.06	0.25	0.22	0.92	0.92	0.05	-0.20	-0.04	0.36	0.83	0.88
	28	0.44	0.15	0.08	0.48	0.67	0.80	0.08	0.05	0.31	0.47	0.66	0.88
	31	-0.09	0.30	0.11	0.21	0.89	0.73	-0.22	-0.14	-0.13	0.52	0.62	0.35
	34	0.61	-0.06	0.03	0.44	0.65	0.98	0.08	-0.09	0.45	0.45	0.63	0.89
	38	-0.07	0.31	0.03	0.17	0.91	0.92	-0.24	-0.05	-0.10	0.42	0.74	0.75

		*Loadings in the (*-*0.15/+0.15) hyperplane: 56 (47.9%)*						*Loadings in the (*-*0.15/+0.15) hyperplane: 64 (54.7%)*					
		*Kaiser-Cerny hyperplane count: 64 (54.7%)*						*Kaiser-Cerny hyperplane count: 62 (53.0%)*					
		*Kaiser-Cerny hyperplane values: 0.19; 0.18; 0.21*						*Kaiser-Cerny hyperplane values: 0.18; 0.12; 0.15*					
		*Ideal hyperplane count: 78 (66.7%)*						*Ideal hyperplane count: 78 (66.7%)*					

*^∗^Subsample 1. Characteristics of the polychoric matrix: determinant <0.001; KMO = 0.85; Bartlett’s statistic = 3,585.80 (df = 741; p < 0.001): Mardia’s statistic = 33.99 (p < 0.001). r, reversed item. **λ**, factorial weight. S-L, second-order Schmid–Leiman approach. δ, uniqueness; IFS, index of factorial simplicity; G, general factor of the exploratory bifactor model. ^†^Not applied to the general factors*.

**Table 5:** Fit indices of the exploratory and confirmatory factor analyses and invariance.

Group/model	χ^2^	df	χ^2^/df	CFI	RMSEA (90% CI)	ΔCFI	ΔRMSEA
**EFA (subsample 1)**							
Long 3-correlated-factors	964.34	627	1.54	0.974	0.042 (0.039-0.045)		
Long bifactor	775.91	591	1.32	0.982	0.032 (0.029-0.035)		
Short 1-advised factor	265.41	54	4.92	0.896	0.112 (0.109-0.115)		
Short 3-forced factors	38.90	33	1.18	0.996	0.024 (0.021-0.037)		
Short 3-forced bifactor	15.38	24	0.64	0.999	0.010 (0.007-0.013)		
New-short 3-correlated factors	47.46	33	1.44	0.993	0.038 (0.035-0.041)		
New-short bifactor	28.08	24	1.17	0.998	0.023 (0.020-0.026)		
**CFA (subsample 2)**							
One-factor (reference)	415.35	54	7.69	0.618	0.147 (0.134-0.160)		
Three-correlated-factors	101.07	51	1.98	0.947	0.056 (0.040-0.072)		
One second-order factor	101.07	51	1.98	0.947	0.056 (0.040-0.072)		
G-Affective bifactor	94.72	46	2.06	0.949	0.058 (0.042-0.075)		
G-Cognitive bifactor	114.91	46	2.50	0.927	0.069 (0.054-0.085)		
G-Reflective bifactor	85.27	46	1.85	0.959	0.052 (0.035-0.070)		
**INVARIANCE (total sample)**							
Three-correlated factors	149.35	51	2.93	0.944	0.056 (0.45-0.066)		
**Subsample (*n*_1_ vs. *n*_2_)**							
Config.	299.2	102	1.95	0.945	0.039 (0.031-0.047)	–^a^	–^a^
Metric	204.1	111	1.84	0.947	0.037 (0.029-0.045)	+0.002	-0.002
Scalar	212.6	123	1.73	0.949	0.034 (0.026-0.042)	+0.002	-0.003
Strict	234.7	135	1.74	0.944	0.034 (0.027-0.042)	-0.005	0.000
Covar.	251.0	141	1.78	0.938	0.035 (0.028-0.042)	-0.006	+0.001
**Age (<55 vs. ≥55 years)**							
Config.	187.3	102	1.84	0.950	0.037 (0.029-0.045)	–^a^	–^a^
Metric	191.9	111	1.73	0.950	0.035 (0.026-0.043)	0.000	-0.002
Scalar	225.9	123	1.84	0.939	0.037 (0.029-0.045)	-0.011	+0.002
Strict	252.5	135	1.88	0.930	0.038 (0.031-0.045)	-0.009	+0.001
Covar.	268.1	141	1.90	0.925	0.039 (0.031-0.046)	-0.005	+0.001
**Sex (male vs. female)**							
Config.	203.4	102	1.99	0.942	0.040 (0.032-0.048)	–^a^	–^a^
Metric	207.1	111	1.87	0.945	0.037 (0.029-0.045)	+0.003	-0.003
Scalar	247.6	123	2.01	0.932	0.041 (0.033-0.048)	-0.013	+0.004
Strict	260.5	135	1.93	0.929	0.039 (0.032-0.046)	-0.003	-0.002
Covar.	265.5	141	1.89	0.929	0.038 (0.031-0.045)	0.000	-0.001
**Studies (second. vs. univ.)**							
Config.	185.7	102	1.82	0.952	0.036 (0.028-0.045)	–^a^	–^a^
Metric	193.9	111	1.75	0.952	0.035 (0.026-0.043)	0.000	-0.001
Scalar	216.4	123	1.76	0.946	0.035 (0.027-0.043)	-0.006	0.000
Strict	249.5	135	1.85	0.934	0.037 (0.030-0.044)	-0.012	+0.002
Covar.	267.3	141	1.90	0.927	0.038 (0.031-0.045)	-0.007	+0.001

*χ^*2*^, chi square; df, degrees of freedom; CFI, comparative fit index; RMSEA, root mean square error of approximation; 90% CI, 90% confidence interval; ΔCFI, variation in CFI from the previous least restrictive model; ΔRMSEA, variation in RMSEA from the previous least restrictive model; Config., configurational invariance; metric, metric invariance; scalar, scalar invariance; strict, strict invariance; Covar., invariance of covariances nested to strict invariance; ^*a*^least restrictive model as a reference; ‘Second. vs. Univ.’, secondary or lower vs. university level of studies*.

The loadings in a Schmid–Leiman general factor, which maintained the same explained variance and fit that the three-correlated factors model, are shown in **Table 4**. A general factor by means of exploratory bifactor analysis improved the fit indices (**Table 5**) and the percentage of explained variance (43.3%). FDI values remained ≥0.91; marginal reliability was ≥0.84 in all the factors; and construct replicability was good in the general factor (*H*_G_ = 0.90), and appropriate but fair in the rest (*H*_1_ = 0.74, *H*_2_ = 0.81, *H*_3_ = 0.77). The ECV and MIREAL values were 0.49 and 0.22, respectively. General simplicity was worsened (IFS = 0.85; SFI = 0.69; Bentler = 0.96), as was the problem of item loadings out of the corresponding theoretical factor (**Table 5**), with only 15 out of 39 loading where they corresponded (38.5%), and with inacceptable factorial SFI values (all of them ≤0.75).

#### Short 3D-WS

In view of this, we explored the possibility of improving the model by discarding certain items. Firstly, the original short SD-WS version (**Table 6**) was explored by means of EFA. Results of the BIC dimensionality test advised a 1-factor solution (**Table 3**), which was not in line with the theoretical background, it only explained 35.4% of the variance and did not show adequate fit (**Table 5**). Therefore, we explored a forced 3-factor solution (explaining 53.9% of the variance), which presented a better fit to the data in addition to good general simplicity (IFS = 0.93; SFI = 0.91; Bentler = 0.98) and SFI values (SFI_1_ = 0.83; SFI_2_ = 0.91; SFI_3_ = 0.99). However, this solution had poor interpretability - only 7 out of 12 items (58.3%) loaded onto their theoretical factor. Factor determinacy values (FDI_1_ = 0.86; FDI_2_ = 0.87; FDI_3_ = 0.96) and marginal reliability (*F*_1_ = 0.74; *F*_2_ = 0.76; *F*_3_ = 0.92) were appropriate, but construct replicability did not reach acceptable values in the first and second components (*H*_1_ = 0.65, *H*_2_ = 0.67, *H*_3_ = 0.87). The inter-factor correlations were moderately high (‘reflective-affective’ ϕ = 0.58, ‘reflective–cognitive’ ϕ = 0.42, ‘affective–cognitive’ ϕ = 0.56).

**Table 6:** Exploratory factor analyses of the Spanish short and new short 3D-WS^∗^.

		Short three-correlated-factors						Short exploratory bifactor					
Factor	item	λ_1_	λ_2_	λ_3_	S-L	δ	IFS^†^	λ_1_	λ_2_	λ_3_	*G*	δ	IFS^†^
Cognitive	22	0.21	0.05	0.38	0.43	0.71	0.66	0.04	-0.06	-0.17	0.60	0.60	0.77
	25	0.49	-0.12	0.02	0.22	0.81	0.91	0.29	-0.26	0.09	0.27	0.83	0.43
	31	0.61	-0.02	-0.08	0.31	0.68	0.97	0.75	-0.30	0.21	0.21	0.46	0.71
	34	0.09	-0.10	0.61	0.37	0.64	0.93	0.02	0.04	-0.41	0.49	0.60	0.98
Affective	12	-0.19	0.55	0.06	0.40	0.75	0.83	-0.08	0.34	-0.03	0.37	0.77	0.91
	21	-0.03	0.62	-0.02	0.51	0.65	0.99	-0.03	0.29	0.11	0.53	0.61	0.81
	33	0.53	0.18	-0.07	0.45	0.62	0.83	0.33	-0.17	0.20	0.48	0.65	0.42
	36	-0.12	0.17	0.56	0.44	0.61	0.82	0.05	0.36	-0.44	0.39	0.61	0.52
Reflective	23	0.14	0.00	0.59	0.48	0.56	0.92	0.14	0.12	-0.38	0.52	0.56	0.72
	26	-0.22	-0.01	0.91	0.45	0.30	0.92	0.03	0.48	-0.77	0.37	0.25	0.63
	29	0.06	-0.02	0.78	0.54	0.36	0.99	0.22	0.28	-0.58	0.48	0.35	0.59
	32	0.07	0.50	0.02	0.49	0.70	0.97	0.34	0.42	0.05	0.30	0.53	0.52

		*Loadings in the (*-*0.15/+0.15) hyperplane: 19 (52.8%)*						*Loadings in the (*-*0.15/+0.15) hyperplane: 14 (38.9%)*					
		*Kaiser-Cerny hyperplane count: 20 (55.6%)*						*Kaiser-Cerny hyperplane count: 18 (50.0%)*					
		*Kaiser-Cerny hyperplane values: 0.19; 0.13; 0.25*						*Kaiser-Cerny hyperplane values: 0.15; 0.24; 0.24*					
		*Ideal hyperplane count: 24 (66.7%)*						*Ideal hyperplane count: 24 (66.7%)*					

		**New short three-correlated-factors**						**New short exploratory bifactor**					

Reflective	23	0.68	0.00	0.13	0.55	0.43	0.95	-0.09	0.42	-0.07	0.70	0.33	0.90
	26	0.81	-0.03	-0.08	0.44	0.43	0.98	-0.09	0.74	0.17	0.32	0.31	0.91
	29	0.78	-0.06	0.01	0.47	0.43	0.99	-0.04	0.64	0.19	0.40	0.42	0.88
	37	0.76	0.09	-0.04	0.53	0.39	0.98	-0.20	0.48	0.03	0.65	0.34	0.78
Cognitive	5	-0.06	0.77	-0.07	0.45	0.49	0.98	-0.13	-0.14	0.58	0.42	0.49	0.85
	7	0.15	0.60	0.04	0.55	0.50	0.91	-0.02	0.07	0.53	0.47	0.49	0.97
	13	-0.04	0.68	-0.07	0.40	0.60	0.98	-0.09	-0.09	0.54	0.34	0.60	0.92
	15	-0.09	0.82	0.03	0.53	0.37	0.98	-0.07	-0.18	0.59	0.52	0.37	0.86
Affective	17r	-0.04	-0.12	0.66	0.41	0.65	0.95	0.62	0.07	0.12	0.25	0.50	0.93
	21r	0.04	0.09	0.44	0.43	0.74	0.93	0.32	0.06	0.18	0.34	0.73	0.64
	24	0.09	0.01	0.59	0.53	0.58	0.97	0.35	0.02	0.03	0.56	0.58	0.98
	33	-0.07	-0.06	0.67	0.44	0.63	0.97	0.40	-0.10	-0.04	0.47	0.62	0.90

		*Loadings in the (*-*0.15/+0.15) hyperplane: 24 (66.7%)*						*Loadings in the (*-*0.15/+0.15) hyperplane: 19 (52.8%)*					
		*Kaiser-Cerny hyperplane count: 24 (66.7%)*						*Kaiser-Cerny hyperplane count: 23 (63.9%)*					
		*Kaiser-Cerny hyperplane values: 0.22; 0.18; 0.17*						*Kaiser-Cerny hyperplane values: 0.17; 0.20; 0.20*					
		*Ideal hyperplane count: 24 (66.7%)*						*Ideal hyperplane count: 24 (66.7%)*					

*^∗^Subsample 1. Characteristics of the short 3D-WS polychoric matrix: Determinant = 0.102; KMO = 0.82; Bartlett’s statistic = 698.80 (df = 66, p < 0.001); Mardia’s statistic = 15.57 (p < 0.001). Characteristics of the new short 3D-WS polychoric matrix: Determinant = 0.057; KMO = 0.80; Bartlett’s statistic = 876.00 (df = 66, p < 0.001); Mardia’s statistic = 15.96 (p < 0.001). r, reversed item; λ, factorial weight; S-L, second-order Schmid–Leiman approach; δ, uniqueness terms; IFS, index of factorial simplicity; G, general factor of the exploratory bifactor model. ^†^Not applied to the general factors*.

The loadings in a general factor by means of the Schmid–Leiman solution and exploratory bifactor analysis can be seen in **Table 6**. Exploratory bifactor analysis improved the fit (**Table 5**) and explained variance (61.7%), although worsening general (IFS = 0.65; SFI = 0.61; Bentler = 0.40), factorial (SFI_1_ = 0.59; SFI_2_ = 0.17; SFI_3_ = 0.91) and individual (**Table 6**) simplicity, and making interpretation difficult due to the appearance of negative weights in the third factor. FDI values (FDI_1_ = 0.75; FDI_2_ = 0.74; FDI_3_ = 0.88; FDI_G_ = 0.87) and marginal reliability estimates (*F*_1_ = 0.56; *F*_2_ = 0.54; *F*_3_ = 0.78; *F*_G_ = 0.76) were insufficient for the first and second factors, and construct replicability values were not acceptable (all of them *H* ≤ 0.70). MIREAL presented a value of 0.24, but ECV was 0.43.

#### New Short 3D-WS

Considering the observed limitations, we explored a new operational definition of 3D-WS by sorting factorial weights into the corresponding theoretical factor. The 12 selected items can be seen in **Table 6**. The fit of the EFA (**Table 5**) for the advised 3-dimensional model (**Table 3**), which explained 60.9% of the variance, as well as the general (IFS = 0.97; SFI = 0.98; Bentler = 0.99), factorial (SFI_1_ = 0.98; SFI_2_ = 0.98; SFI_3_ = 0.98) and individual (**Table 7**) simplicity values was adequate, with all the items loading onto their corresponding factor. Factor determinacy (FDI_1_ = 0.95; FDI_2_ = 0.93; FDI_3_ = 0.91), marginal reliability (*F*_1_ = 0.90; *F*_2_ = 0.87; *F*_3_ = 0.83) and construct replicability (*H*_1_ = 0.86, *H*_2_ = 0.83, *H*_3_ = 0.74) were appropriate. The inter-factor correlations were moderately high (‘reflective-affective’ ϕ = 0.51, ‘reflective–cognitive’ ϕ = 0.46, ‘affective–cognitive’ ϕ = 0.55).

**Table 7:** Confirmatory factor analysis of the new short three-correlated-factors 3D-WS^†^.

Factor	Item	*Mn*	*SD*	Skew	Kurt	Item-rest (*f*)	Item-rest (*t*)	λ	δ
Reflective	23	3.94	1,10	-0.91	0.10	0.68	0.62	0.85^∗^	0.28
	26	3.65	1.13	-0.51	-0.50	0.67	0.63	0.77^∗^	0.41
	29	3.53	1.16	-0.52	-0.50	0.60	0.62	0.71^∗^	0.50
	37	3.91	1.13	-0.93	0.12	0.59	0.64	0.75^∗^	0.44
Cognitive	5	3.82	1.00	-0.68	-0.10	0.55	0.53	0.70^∗^	0.51
	7	4.40	0.86	-1.82	3.69	0.59	0.45	0.76^∗^	0.42
	13	3.52	1.13	-0.60	-0.34	0.55	0.46	0.68^∗^	0.54
	15	4.20	0.99	-1.47	2.04	0.66	0.55	0.87^∗^	0.24
Affective	17r	4.28	0.68	-0.46	-0.50	0.39	0.38	0.58^∗^	0.66
	21r	3.79	1.03	-0.68	-0.10	0.31	0.32	0.51^∗^	0.73
	24	4.03	1.05	-1.20	1.07	0.37	0.37	0.63^∗^	0.60
	33	4.06	0.98	-1.18	1.27	0.39	0.38	0.64^∗^	0.59

*^†^Subsample 2. Characteristics of the polychoric matrix: Determinant = 0.038; KMO = 0.80; Bartlett’s statistic = 997.20 (df = 66, p < 0.001); Mardia’s coefficient = 18.25 (p < 0.001). Mn, mean; SD, standard deviation; skew, skewness; kurt, kurtosis; item-rest, discrimination coefficient for the factor (f) and total (t). λ, factorial weight; δ, uniqueness; r, reversed item; ^∗^p < 0.001*.

The loadings in a Schmid–Leiman general factor solution and exploratory bifactor analysis are shown in **Table 6**. The exploratory bifactor analysis improved the model fit (**Table 5**), explaining 67.8% of the variance. Factorial simplicity remained adequate at the general (IFS = 0.89; SFI = 0.89; Bentler = 0.98), factorial (SFI_1_ = 0.78; SFI_2_ = 0.94; SFI_3_ = 0.91) and individual (**Table 6**) levels, with all the items loading onto the corresponding factor. FDI was not adequate for the third factor (FDI_1_ = 0.90; FDI_2_ = 0.81; FDI_3_ = 0.67; FDI_G_ = 0.95), and marginal reliability was only sufficient for the first and general factors (*F*_1_ = 0.80; *F*_2_ = 0.66; *F*_3_ = 0.44; *F*_G_ = 0.90). Construct replicability was appropriate for the first and general factor, but not for the second and third (*H*_1_ = 0.78, *H*_2_ = 0.59, *H*_3_ = 0.62; *H*_G_ = 0.94). ECV and MIREAL values were 0.52 and 0.29, respectively.

### Confirmatory Factor Analyses

#### New Short 3D-WS

The descriptive of the new short 3D-WS proposal (subsample 2), are shown in **Table 7**. The BIC dimensionality test showed a 3-factor solution (**Table 3**), explaining 63.8% of the variance. The CFA for a 3-correlated factors solution showed adequate loadings, ranging from 0.51 to 0.87 (**Table 8**), and presented adequate fit without introducing covariances between the errors (**Table 5**). Uniqueness was similar to that obtained from EFA using subsample 1, and residual covariances were low and equally distributed among all the items -average absolute value = 0.04. FDI values were adequate (FDI-reflective = 0.78; FDI-cognitive = 0.87; FDI-affective = 0.80). Construct replicability was appropriate -a bit fair in the affective factor: H-reflective = 0.87, H-cognitive = 0.86, H-affective = 0.70. The inter-factor correlations were moderate and significant for ‘reflective–cognitive’ (ϕ = 0.43; *p* < 0.001) and ‘reflective–affective’ (ϕ = 0.41; *p* < 0.001), and low but significant between ‘cognitive-affective’ (ϕ = 0.21; *p* < 0.05). The AVE and CR values were adequate in the reflective (AVE = 0.60; ω_S_ = 0.85) and cognitive (AVE = 0.57; ω_S_ = 0.84) factors, although they were fair in the affective component (AVE = 0.36; ω_S_ = 0.69) -the effectiveness of alpha coefficients was corroborated in the reflective (α = 0.82; 95% CI = 0.78-0.85), and cognitive (α = 0.78; 95% CI = 0.73-0.82) components, but it was not in the affective (α = 0.60; 95% CI = 0.52-0.67), showing certain impact of the difference in the size of factorial loadings. Nevertheless, all AVE values were higher than the corresponding squared correlation between factors, which suggested discriminant validity among them. Moreover, CR values were >0.60 and with AVE values close to or higher than 0.40, suggesting construct convergent validity.

**Table 8:** Explanatory power of the new short 3D-WS on psychological health-related outcomes.

	DV/IVs	*R*^2^	*Se*	*F*	df	*p*^a^	*r*	*b*	*Se*	Beta	*t*	*p*^b^
Subsample 1												
	**PIL**	0.42	13.78	70.78	3/288	<0.001						
	Reflective						0.63*	1.04	0.10	0.55	10.94	<0.001
	Cognitive						0.25*	0.01	0.09	0.01	0.11	0.916
	Affective						0.41*	0.35	0.09	0.19	3.77	<0.001
	**MSBS**	0.40	28.51	66.42	3/299	<0.001						
	Reflective						-0.62*	-2.14	0.19	-0.56	-11.04	<0.001
	Cognitive						-0.32*	-0.41	0.18	-0.11	-2.22	0.026
	Affective						-0.32*	-0.24	0.19	-0.06	-1.26	0.208
	**PANAS-p**	0.33	6.88	48.00	3/290	<0.001						
	Reflective						0.55*	0.39	0.05	0.46	8.45	<0.001
	Cognitive						0.28*	0.05	0.05	0.05	1.01	0.313
	Affective						0.38*	0.15	0.05	0.18	3.25	0.001
	**PANAS-n**	0.21	5.67	27.89	3/296	<0.001						
	Reflective						-0.45*	-0.30	0.04	-0.46	-7.99	<0.001
	Cognitive						-0.23*	-0.07	0.04	-0.10	-1.79	0.075
	Affective						-0.12^†^	0.07	0.04	0.10	1.82	0.069
	**DERS**	0.47	14.35	86.47	3/287	<0.001						
	Reflective						-0.68*	-1.27	0.10	-0.63	-12.95	<0.001
	Cognitive						-0.30*	-0.11	0.09	-0.05	-1.13	0.261
	Affective						-0.36*	-0.17	0.10	-0.09	-1.77	0.078

Subsample 2												
	**PIL**	0.36	13.84	55.61	3/290	<0.001						
	Reflective						0.59*	0.91	0.09	0.54	10.59	<0.001
	Cognitive						0.26*	0.12	0.09	0.06	1.33	0.916
	Affective						0.29*	0.17	0.09	0.10	1.97	<0.001
	**MSBS**	0.50	26.14	100.13	3/301	<0.001						
	Reflective						-0.70*	-2.31	0.16	-0.66	-14.72	<0.001
	Cognitive						-0.35*	-0.49	0.16	-0.14	-3.12	0.002
	Affective						-0.21*	0.14	0.16	0.04	0.88	0.381
	**PANAS-p**	0.26	7.12	35.19	3/292	<0.001						
	Reflective						0.49*	0.33	0.04	0.42	7.58	<0.001
	Cognitive						0.20*	0.02	0.04	0.02	0.40	0.401
	Affective						0.32*	0.14	0.04	0.18	3.29	0.001
	**PANAS-n**	0.31	5.52	46.25	3/296	<0.001						
	Reflective						-0.56*	-0.38	0.03	-0.59	-11.16	<0.001
	Cognitive						-0.16^‡^	0.01	0.03	-0.01	0.08	0.933
	Affective						-0.13^†^	0.05	0.03	0.08	1.46	0.147
	**DERS**	0.47	13.96	83.34	3/280	<0.001						
	Reflective						-0.68*	-1.21	0.09	-0.65	-13.64	<0.001
	Cognitive						-0.30*	-0.20	0.09	-0.10	-2.23	0.027
	Affective						-0.24*	-0.01	0.09	-0.01	-0.02	0.985

*DV, dependent variable; IVs, independent variables; R^*2*^, adjusted determination coefficient. F, Snedecor’s F. df, degrees of freedom. Se, standard error. p^*a*^, p-value associated with the ANOVA test for the model adjustment. r, Pearson’s raw correlation. b, regression coefficient. Beta, standardized regression coefficient. t, Student’s t-value related to the Wald test on regression coefficients. p^*b*^, p-value associated with the Wald test on regression coefficients. ^∗^p < 0.001, ^‡^p < 0.01, ^†^p < 0.05.*

#### General Factor Structure

**FIGURE 2:**
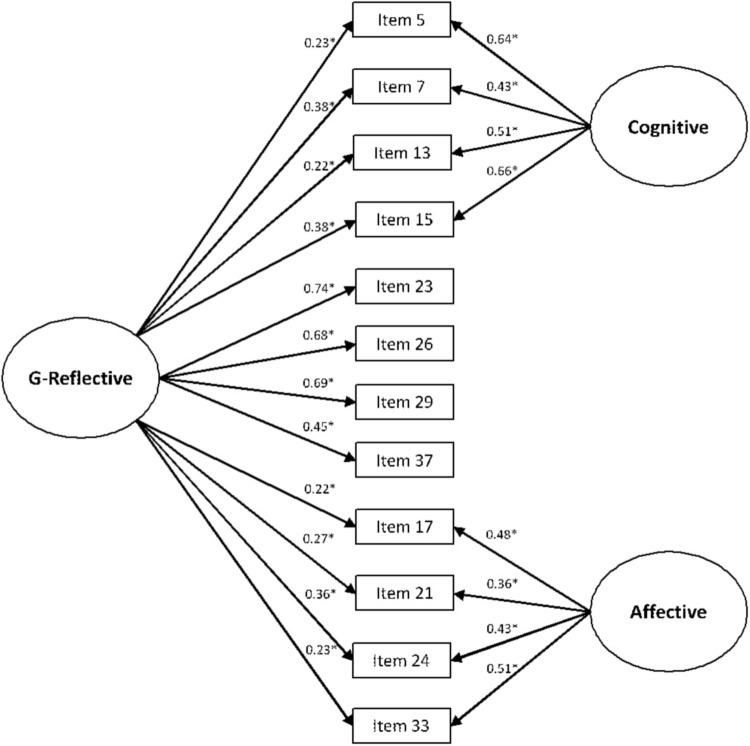
G-reflective bifactor structure for the new proposed short 3D-WS. G-reflective, G-reflective general factor; cognitive, cognitive factor; affective, affective factor. ^∗^*p* < 0.001.

The general factor structure of the new short 3D-WS was tested by CFA and a second-order solution using subsample 2. It presented the same fit as that obtained in the three-correlated-factor model, with high and significant second-order loadings in the reflective (γ-reflective = 0.90, *R*^2^ = 0.81, *p* < 0.001), and moderate and significant in the cognitive (γ-cognitive = 0.48, *R*^2^ = 0.23, *p* < 0.001) and affective (γ-affective = 0.44, *R*^2^ = 0.19, *p* < 0.001). The G-Reflective bifactor structure improved the fit (**Table 5**), showing a G-Reflective general factor with significant loadings in all the items (**Figure 2**). Loadings of the G-Reflective were greater in those items of the reflective theoretical factor (ranging from 0.45 to 0.78), compared to the items from the cognitive and affective components (ranging from 0.22 to 0.38). All the items ranged from 0.16 to 0.76 in the one-factor solution taken as a reference, which showed poor fit (**Table 5**). We also explored the fit of an affective bifactor structure (in which the general factor would incorporate the affective traits), and a cognitive bifactor structure (in which the general factor would incorporate the cognitive traits), but both the G-Affective and G-Cognitive general factor solutions showed worse fit to the data than the G-Reflective (**Table 5**). Moreover, a bifactor solution maintaining the reflective, affective and cognitive factors at the same orthogonal level did not reach identification. The CR for the total scale was ω = 0.81 -alpha coefficient was α = 0.77; 95% CI = 0.72-0.81, and removing any item did not improve this value-, with ω_H_ = 0.60. Therefore, almost 3/4 of the reliable variance in total scores came from the G-reflective. The subscale score variance after controlling for the effects of the G-Reflective was ω_HS_ = 0.58 for the cognitive (2/3 of the reliable variance in the cognitive factor were out of the influence of the G-Reflective), and ω_HS_ = 0.45 for the affective (2/3 of the reliable variance in the affective factor were out of the influence of the G-Reflective). FD values were rather fair (FD-G-Reflective = 0.78, FD-cognitive = 0.76, FD-affective = 0.66), but the H index was only appropriate for the G-Reflective (H-G-Reflective = 0.80, H-cognitive = 0.67, H-affective = 0.51). The ECV was 0.53, reflecting that common variance was equally spread across the G-Reflective and the other two factors (i.e., affective, cognitive). The PUC was 0.67 (around 2/3 of correlations informed directly on the general factor). All these results suggested that common variance should not be regarded as essentially unidimensional.

#### Invariance Analyses

The three-correlated-factor model was used to explore the invariance of the new short 3D-WS across subsamples (*n*_1_ = 312 vs. *n*_2_ = 312), age (<55 years = 515 vs. ≥55 years = 109), sex (female = 472 vs. male = 152) and studies (primary/secondary = 128 vs. university = 496). CFA showed appropriate fit in the overall sample (**Table 5**). None of the increasingly restrictive nested models of invariance exceeded both cut-off recommendations (e.g., ΔCFI and ΔRMSEA) at the same time with respect to subsample, sex, age and level of studies (**Table 5**), providing a reasonable level of approximate fit to the data. There were no differences when comparing latent means according to subsample (reflective: ΔMn = -0.01, *p* = 0.846, *d* = 0.01; affective: ΔMn = -0.01, *p* = 0.703, *d* = 0.03; cognitive: ΔMn = -0.07, *p* = 0.207, *d* = 0.10). However, there were differences according to age, with higher values in the older group in the reflective (ΔMn = 0.27, *p* = 0.003, *d* = 0.35) and affective (ΔMn = 0.13, *p* = 0.010, *d* = 0.38), but not in the cognitive (ΔMn = 0.02, *p* = 0.845, *d* = 0.03). There were also differences according to sex, with lower values in females in the reflective (ΔMn = -0.18, *p* = 0.037, *d* = 0.22), and higher values in the affective (ΔMn = 0.10, *p* = 0.024, *d* = 0.30), but there were no differences in the cognitive (ΔMn = 0.06, *p* = 0.414, *d* = 0.09). There were differences according to education level, with higher values for having university studies in the cognitive component (ΔMn = 0.21, *p* = 0.008, *d* = 0.33), but there were no differences in the reflective (ΔMn = 0.18, *p* = 0.051, *d* = 0.22) and affective (ΔMn = 0.01, *p* = 0.874, *d* = 0.02) factors.

### Convergence and Explanatory Power

The raw correlations between the same dimension of the original long and new proposed short 3D-WS using EAP factor scores were significant (all of them *p* < 0.001), and high [subsample 1: reflective *r* = 0.84 (adj-*r* = 0.68), cognitive: *r* = 0.87 (adj-*r* = 0.67), affective: *r* = 0.87 (adj-*r* = 0.55); subsample 2: reflective *r* = 0.84 (adj-*r* = 0.69), cognitive *r* = 0.88 (adj-*r* = 0.72), affective *r* = 0.82 (adj-*r* = 0.52)].

In general, all factors of the new short 3D-WS showed high and significant raw associations with all the psychological health-related outcomes in both subsamples (**Table 8**) -the cognitive and affective components presented small but significant associations with PANAS-negative. The reflective component had the highest raw associations with all the psychological outcomes in both subsamples. The explanatory power of the new short 3D-WS in relation to all psychological variables was high and significant, and it was similar across subsamples, ranging from *R*^2^ = 0.21 (PANAS-negative, subsample 1) to *R*^2^ = 0.50 (boredom, subsample 2). The fit of the regression models was adequate (all of them *p* < 0.001). Judging by the standard errors, the models showed similar predictions across subsamples. Consistently, the reflective factor contributed to explaining all the psychological variables considered, with Beta absolute values ≥0.42 (*p* < 0.001). The affective factor contributed to explain purpose in life -subsample 1: Beta = 0.19 (*p* < 0.001); subsample 2: Beta = 0.10 (*p* = 0.048)- and positive affectivity -Beta = 0.18 (*p* = 0.001) in both subsamples. The cognitive factor contributed to explaining boredom in both subsamples -subsample 1: Beta = 0.11 (*p* = 0.026); subsample 2: Beta = 0.10 (*p* = 0.002). Except for PANAS-negative, residual distributions did not present problems of asymmetry. DW values were ≈2.00 in all the cases. Finally, tolerance and VIF values did not point to collinearity problems [reflective (subsample 1: T ≥ 0.78, VIF ≤ 1.28; subsample 2: T ≥ 0.82, VIF ≤ 1.21), affective (subsample 1: T ≥ 0.79, VIF ≤ 1.26; subsample 2: T ≥ 0.87, VIF ≤ 1.16), cognitive (subsample 1: T ≥ 0.83, VIF ≤ 1.20; subsample 2: T ≥ 0.88, VIF ≤ 1.19)].

## Discussion

### Spanish 3D-WS

The primary purpose of the present study was to validate a Spanish version of the 3D-WS. To the best of our knowledge, there are no robust validations of any questionnaires used to assess the difficult to gauge wisdom construct in the Spanish language. In our study, the factorial structure of the original long 3D-WS (Ardelt, 2003) was not replicated through EFA, and although the three-dimensionality fitted to the data, few items loaded adequately onto their corresponding theoretical factor. Most of the 3D-WS items were not of original design, but were taken from other existing questionnaires from a variety of associated content domains, e.g., Need for cognition (Cacioppo and Richard, 1983), attitudes about reality (Unger et al., 1986), dogmatism (Rokeach, 1960), ambiguity Tolerance (MacDonald, 1970), ideas of reference (Sears, 1937), perspective-taking (Davis, 1980), personal problem-solving (Heppner and Petersen, 1982), resentment (Bachman et al., 1967), empathy (Mehrabian and Epstein, 1972), acceptance of others (Fey, 1955), compassion (Beutel and Marini, 1995), empathic concern (Davis, 1980), helping disposition (Severy, 1975), overt but safe aggression (Webster et al., 1955), Liking People (Filsinger, 1981), acceptance of self and others (Shaw and Wright, 1967). The fact that the 3D-WS items cover a large range of personality characteristics attempting to capture a broad essence of wisdom (Ardelt, 2003), might be why many of them did not load strongly onto their expected factor, displaying a complex cross-loading structure. Despite this, factor determinacy, marginal reliability and construct replicability were acceptable, showing a moderately high pattern of inter-factor correlations which, nonetheless, explained a low percentage of variance. The Schmid–Leiman solution showed moderate factorial weights for the second-order factors, and an exploratory bifactor structure improved the fit to the data, but the explained common variance suggested a weak common factor, and the problem of cross-loadings remained.

The original short 3D-WS factorial structure (Thomas et al., 2017) presented greater difficulties because it was necessary to force the theoretical three-dimensional structure through EFA. The factor determinacy and marginal reliability of this solution were appropriate, explaining a considerable percent of the total variance, with moderately high inter-factorial correlations and adequate fit indices. However, construct replicability did not reach acceptable values, and only half of the items loaded where they corresponded. The loadings in the Schmid–Leiman approach were moderate, and the bifactor solution improved the fit and the percentage of explained variance -but simplicity, determinacy, marginal reliability and construct replicability were not adequate, with the appearance of negative loadings that hindered interpretability. Nevertheless, explained common variance was insufficient, and thus, the original short 3D-WS could not be considered as minimally acceptable.

In this context, it was necessary to explore a new 3D-WS proposal using those items with the best factorial behavior, which could provide a reasonable solution in terms of adjustment, simplicity and possibilities of interpretation. This was not the first time that the 3D-WS had to be adapted; for example, the Korean 3D-WS showed a distinct factor structure and item content as a result of adding culturally specific factors of wisdom such as modesty and unobtrusiveness (Kim and Knight, 2014). Thus, 12 items were selected, four in each dimension, representing wisdom in a more concise way, but in accordance with the original proposal (Ardelt, 2000, 2003). The reflective factor was operationalized as the ability to overcome self-centredness and subjectivity (e.g., “Sometimes I get so charged up emotionally that I am unable to consider many ways of dealing with my problems,” -reversed). The cognitive was defined as the ability to understand the ambiguity of human nature and the limits of knowledge (e.g., “People are either good or bad,” -reversed). The affective included the presence of sympathetic and compassionate behaviors toward others (e.g., “I don’t like to get involved in listening to another person’s troubles,” -reversed). This new proposal overcame the previous limitations, showing a three-factor structure with moderately high inter-factor correlations and adequate fit. All the items loaded where they corresponded, and an important percentage of the variance was explained. Moreover, simplicity was very good, as was determinacy, suggesting the factor score estimates unambiguously reflected the latent levels they attempted to estimate (Beauducel, 2011). Marginal reliability was acceptable, as was construct replicability, which suggested all the factors were well defined (Hancock and Mueller, 2000; Brown and Croudace, 2015; Rodriguez et al., 2016). The loadings in the Schmid–Leiman approximation and the exploratory bifactor analysis were moderately high, and the latter improved the model fit and the percent of variance explained, maintaining factorial simplicity and item distribution. However, factor determinacy, marginal reliability and construct replicability were not sufficient in all the components, with a general factor presenting an explained common variance of around 50%.

The new short three-correlated-factor model was also tested on a second subsample using CFA, obtaining adequate loadings and fit indices. Likewise, factor determinacy and construct replicability were appropriate, as were the average variance extracted and composite reliability, and although these two were a bit fair in the affective component -something that well deserves to be the subject of future research-, it was possible to establish convergent and discriminant validity following the Fornell and Larcker (1981) criteria. Interestingly, inter-factor correlations presented the same pattern as that obtained in the original design of the long 3D-WS (Ardelt, 2003), with the reflective component showing moderately high relationships with the other two (i.e., cognitive, affective), which in comparison were less associated between them.

### General Factor

The existence of a possible general wisdom factor was firstly explored by means of the Schmid–Leiman approach and exploratory bifactor analysis using EFA, as explained above. After that, it was explored by a second-order solution using CFA and subsample 2, which demonstrated the same fit to the data as that of the three-correlated-factor solution. So far, the idea of a G-Reflective general factor that would explain the possible influences of the reflective traits on all the wisdom aspects was supported by: (a) the three-factor structure of data suggested by the dimensionality test; (b) the differential strength of inter-factor correlations in the first-order approach -with higher values when the reflective component was implied; and (c) a loading structure from the second-order general factor with values four times greater toward the reflective component, compared with the others. Thus, the G-Reflective structure was tested in these conditions by means of CFA and the bifactor model using subsample 2, achieving better fit than previous models and obtaining significant loadings from the G-Reflective onto all the items -with higher values for the specific reflective items, highlighting the general reflective tendency. The other alternative bifactor models showed worse fit or did not reach identification. Therefore, the G-Reflective bifactor model was accepted as the best approach to the general factor for the new short 3D-WS. This G-Reflective accounted for around 75% of the reliable variance in total scores. The percent of reliable variance in subscales due to the effects of the G-Reflective was roughly 33%. The factor determinacy and construct replicability for the G-Reflective were fair, but they were insufficient for the other two subscales. About one-half of explained common variance was attributable to the G-Reflective general factor. Therefore, it was equally split into two sources, the G-Reflective, and the cognitive and affective components. Thus, the new short 3D-WS should not be considered primarily unidimensional in the context of uncontaminated correlations found (Rodriguez et al., 2016), although the G-Reflective factor was a relevant source of explained common variability.

### Invariance

Multi-group CFA demonstrated strict invariance -including the level of covariances between latent components- for the three-correlated-factor solution of the new short 3D-WS with respect to subsample, and three important factors that might be related to wisdom, such as age, sex and study level (Ardelt, 2000, 2003; Thomas et al., 2017). This result provides support for the possibilities of generalizing the described assessment model for wisdom. As expected, random subsampling did not determine differences in the latent means of the wisdom factors. However, those participants ≥55 years old showed higher levels in the reflective and affective factors, with moderately low effects. Wisdom does not need to increase automatically with age (Ardelt, 1997; Staudinger, 1999; Webster, 2003), because the development of wisdom would require time but also active experience in overcoming subjectivity and projections (Kekes, 1983; Ardelt, 2003). However, it is accepted that wisdom seems to reach a maximum peak at around the mid-50s, usually being higher when this group is compared to younger people, although it might show a decrease in the final stage of life, like other capabilities (Wink and Helson, 1997; Ardelt, 2011; Webster et al., 2014). On the other hand, female participants showed lower levels in the reflective factor, but higher values in the affective, with low and moderately low effects, respectively. In a previous study, differences were found in wisdom according to gender, with females presenting lower scores in the cognitive factor (Ardelt, 2003). These differences were attributed to the fact that men have usually been more encouraged to develop their cognitive capacities, and to know the deeper meaning of phenomena and events than women have, and maybe this could be extensive to their reflective capacities. Likewise, and particularly in the Spanish context, women have traditionally assumed the principal role of providing informal care (García-Calvente et al., 2004), and this gender-work identity may facilitate experiences so that they develop higher levels of emotional wisdom. Finally, a university study level was related to higher levels in the cognitive factor with a moderately low effect, compared to secondary and lower levels of education. It has been said that individuals in search of wisdom are more likely to pursue more advanced educational levels (Ardelt, 2003), and the same could be applied here, but the cross-sectional nature of this study makes causality difficult to establish.

### Explanatory Power

The new short 3D-WS proposal was convergent with the original long version of the scale (which although not entirely recommended because of its weak factorial structure, was taken into account as a content reference). The new short 3D-WS was highly associated with all psychological health-related variables in the expected directions using multivariate regression models, highlighting the relevance of wisdom in terms of mental health and general well-being, similar to other studies (Ardelt, 1997, 2000, 2003; Le, 2011; Bergsma and Ardelt, 2012; Jeste et al., 2013; Roháriková et al., 2013; Zacher et al., 2013; Webster et al., 2014; Thomas et al., 2017). The reflective component was the most powered wisdom factor to explain well-being across both subsamples, and it was significantly related to all psychological health-related variables when controlling the other wisdom components. This is congruent with the idea that an ego-decentred mindset enables wise thinking and behavior regarding personally meaningful issues (Grossmann, 2017). It therefore reinforces the idea of the reflective component as the core dimension of wisdom from which the other wisdom components may be developed (Ardelt, 2003; Thomas et al., 2017). Interestingly, the cognitive factor contributed to inversely explain boredom (adverse outcome), an important outcome that is related to distinct mental disorders (Alda et al., 2015), whereas the affective factor contributed to explain positive affectivity and purpose in life (favorable outcomes), which is in line with the definition and valence of these wisdom dimensions.

### Strengths and Limitations

As strengths, we highlight the large sample size used, which enabled us to properly develop the statistical analyses by dividing the total group into two randomly selected subsamples in which to independently perform the exploratory and confirmatory analyses. In addition, the basic assumptions for the type of data analysis utilized were accepted, although the distribution of residuals was not normal in the case of negative affect, which may have affected the confidence intervals, but with minor consequences as a result of the large sample size used. Nevertheless, the present study has several limitations. First, the sample selection procedure used obligated us to be cautious when generalizing our results; therefore, this work should be considered a heuristic guide to drive future research. Second, because this work was oriented in extension rather than in depth, as a result of the early and preliminary stage in the development of a complex, difficult-to-grasp construct but with promising perspectives (Staudinger and Glück, 2011), the relationships between wisdom and the other health-related constructs were established using general regression models, which did not consider measurement errors. Third, the use of a cross-sectional design did not enable us to drive the analysis toward testing causal hypotheses; it only allowed us to speak in terms of relationships and explanatory power. Fourth, we did not employ other alternative conceptualizations of wisdom (Taylor et al., 2011) to assess the convergent validity of the model evaluated as a result of the absence of appropriate measures in Spanish. Future studies that aim to develop and refine the wisdom construct should take into account and solve this limitation. Finally, the instruments were always presented in the survey in the same order, and therefore, their presentation was not counterbalanced to control for order effects. Likewise, the self-report technique employed may carry a certain response bias in terms of acquiescence or social desirability (Taylor et al., 2011), as specifically indicated when studying the field of personality (Navarro-González et al., 2016), thus attenuating the results. For these reasons, and depending on the focus of the research, peer ratings should also be taken into account as a suitable measure of wisdom (Redzanowski and Glück, 2013).

## Conclusion

The new short 3D-WS appeared to be a reliable instrument for measuring wisdom in the Spanish general population through its reflective, cognitive, and affective components. It has the advantage of gaining brevity, though perhaps at the expense of reducing the extension of the construct. The reflective trait seemed to influence the others by a general reflective factor which, nevertheless, did not acquire enough strength as to be taken separately in the measurement model. Wisdom may be considered relevant for its relationships with other psychological health-related outcomes associated with well-being and mental disorders. The present work will facilitate the conceptualization, measurement, and development of future psychological knowledge and research regarding wisdom. The study of specific samples, such as other culturally different Spanish-speaking subjects (from Central or South America), should determine the extent to which the Spanish new short 3D-WS is replicable across such contexts.

## Ethics Statement

The study protocol was approved by the ethical review board of the regional health authority of Aragon, Spain (PI16/0117). All participants submitted a written informed consent form online attesting to their willingness to participate in the study.

## Author Contributions

JG-C and JM-M conceived and designed the study and wrote the manuscript. LB performed the online survey and prepared the database. JM-M analyzed the data. All authors performed a critical revision of the manuscript and accepted the final version.

## Conflict of Interest Statement

The authors declare that the research was conducted in the absence of any commercial or financial relationships that could be construed as a potential conflict of interest.

## Footnotes

1 www.surveymonkey.com
