# Supplementary material for: Exploring the Wisdom Structure: Validation of the Spanish New Short Three-Dimensional Wisdom Scale (3D-WS) and Its Explanatory Power on Psychological Health-Related Variables
Source: Front Psychol. 2018 May 14;9:692. doi: 10.3389/fpsyg.2018.00692 (PMC5960699; doi:10.3389/fpsyg.2018.00692)
Supplement: Supplementary file 1 [file Data_Sheet_1.docx]

**Additional File 1: Spanish version of the Three-Dimensional Wisdom Scale (3D-WS)**

| 1. En nuestro complicado mundo, la única manera en que podemos saber lo que está pasando es fiarse de los líderes o expertos en los que se pueda confiar [C] |
| --- |
| 2. Me molestan las personas que no son felices y que sólo sienten lástima por sí mismos. [A] |
| 3. La vida es básicamente la misma la mayor parte del tiempo [C] |
| 4. La gente sobrestima los sentimientos y sensibilidad de los animales [A] |
| **5. Se puede clasificar a casi toda la gente como honrada o como no honrada [C]** |
| 6. Me sentiría mucho mejor si mis circunstancias actuales cambiasen [R] |
| **7. Sólo existe una manera correcta de hacer algo [C]** |
| 8. Hay algunas personas que conozco que nunca me gustarán [A] |
| 9. Es mejor no saber demasiado sobre las cosas que no se pueden cambiar [C] |
| 10. A menudo las cosas me salen mal sin que sea culpa mía [R] |
| 11. La ignorancia es una bendición [C] |
| 12. Puedo sentirme cómodo/a con todo tipo de personas (r) [A] |
| **13. Una persona o sabe la respuesta a una pregunta o no la sabe [C]** |
| 14. Realmente no es mi problema si otros están en apuros y necesitan ayuda [A] |
| **15. Las personas son buenas o malas [C]** |
| 16. En un desacuerdo, intento considerar la postura de todas las partes antes de tomar una decisión (r) [R] |
| **17. Si veo a personas en apuros, intento ayudarles de una forma u otra (r) [A]** |
| 18. Cuando estoy molesto/a con alguien, suelo intentar ponerme en su lugar por un tiempo (r) [R] |
| 19. Hay ciertas personas que me desagradan tanto que en mi interior estoy contento cuando son descubiertas y castigadas por algo que hicieron [A] |
| 20. Siempre intento mirar todos los aspectos de un problema (r) [R] |
| **21. A veces siento auténtica compasión por todo el mundo (r) [A]** |
| 22. Trato de anticipar y evitar situaciones en las que es probable que tenga que pensar profundamente sobre algo [C] |
| **23. Cuando miro hacia atrás y veo lo que me ha pasado, no puedo evitar sentir resentimiento [R]** |
| **24. A menudo no he consolado a otra persona cuando lo necesitaba [A]** |
| 25. Me atrae poco un problema si considero que no tiene solución [C] |
| **26. Si las cosas van mal, o me enfado mucho o me deprimo mucho [R]** |
| 27. A veces no me siento muy apenado/a por otras personas cuando tienen problemas [A] |
| 28. A menudo no entiendo el comportamiento de la gente [C] |
| **29. A veces me siento tan cargado/a emocionalmente que soy incapaz de considerar la manera de hacer frente a mis problemas [R]** |
| 30. A veces cuando la gente me habla, deseo que me dejen en paz [A] |
| 31. Prefiero dejar que las cosas ocurran a intentar comprender por qué sucedieron así [C] |
| 32. Cuando estoy confundido/a por un problema, una de las primeras cosas que hago es estudiar la situación y considerar todos los elementos de información relevantes (r) [R] |
| **33. No me gusta implicarme escuchando los problemas de otra persona [A]** |
| 34. Estoy indeciso/a sobre tomar decisiones importantes después de reflexionar sobre ellas [C] |
| 35. Antes de criticar a alguien, intento imaginarme cómo me sentiría yo en su lugar (r) [R] |
| 36. Me irrito fácilmente con las personas que discuten conmigo [A] |
| **37. Cuando miro hacia atrás y veo lo que me ha pasado, me siento engañado/a [R]** |
| 38. Para mí está bien simplemente con conocer la respuesta a un problema, en lugar de entender las razones por las que esa respuesta es adecuada [C] |
| 39. A veces encuentro difícil ver las cosas desde el punto de vista de otra persona [R] |

Escala de respuesta: Totalmente de acuerdo (1), De acuerdo (2), Neutral (3), En desacuerdo (4), Totalmente en Desacuerdo (5)

En negrita se señalan los ítems de la versión abreviada: New short Three-Dimensional Wisdom Scale (3D-WS). (r): ítem reverso.

Las letras entre corchetes pertenecen a las subescalas de la escala original: [A]: Afectiva, [C]: Cognitiva, [R] Reflexiva.
