# Supplementary material for: Exploring the Wisdom Structure: Validation of the Spanish New Short Three-Dimensional Wisdom Scale (3D-WS) and Its Explanatory Power on Psychological Health-Related Variables
Source: Front Psychol. 2018 May 14;9:692. doi: 10.3389/fpsyg.2018.00692 (PMC5960699; doi:10.3389/fpsyg.2018.00692)
Supplement: Supplementary file 2 [file Data_Sheet_2.docx]

**Additional File 2: Original version of the Three-Dimensional Wisdom Scale (3D-WS)**

| 1. In this complicated world of ours, the only way we can know what’s going on is to rely on leaders or experts who can be trusted [C] |
| --- |
| 2. I am annoyed by unhappy people who just feel sorry for themselves [A] |
| 3. Life is basically the same most of the time [C]  4. People make too much of the feelings and sensitivity of animals [A] |
| **5. You can classify almost all people as either honest or crooked [C]** |
| 6. I would feel much better if my present circumstances changed [R]  **7. There is only one right way to do anything [C]**  8. There are some people I know I would never like [A]  9. It is better not to know too much about things that cannot be changed [C]  10. Things often go wrong for me by no fault of my own [R]  11. Ignorance is bliss [C]  12. I can be comfortable with all kinds of people (r) [A]  **13. A person either knows the answer to a question or he/she doesn’t [C]**  14. It’s not really my problem if others are in trouble and need help [A]  **15. People are either good or bad [C]** |
| 16. I try to look at everybody’s side of a disagreement before I make a decision (r) [R] |
| **17. If I see people in need, I try to help them one way or another (r) [A]** |
| 18. When I’m upset at someone, I usually try to “put myself in his or her shoes” for a while (r) [R] |
| 19. There are certain people whom I dislike so much that I am inwardly pleased when they are caught and punished for  something they have done [A]  20. I always try to look at all sides of a problem (r) [R]  **21. Sometimes I feel a real compassion for everyone (r) [A]**  22. I try to anticipate and avoid situations where there is a likely chance I will have to think in depth about something [C] |
| **23. When I look back on what has happened to me, I can’t help feeling resentful [R]** |
| **24. I often have not comforted another when he or she needed it [A]**  25. A problem has little attraction for me if I don’t think it has a solution [C] |
| **26. I either get very angry or depressed if things go wrong [R]**  27. Sometimes I don’t feel very sorry for other people when they are having problems [A]  28. I often do not understand people’s behavior [C]  **29. Sometimes I get so charged up emotionally that I am unable to consider many ways of dealing with my problems [R]**  30. Sometimes when people are talking to me, I find myself wishing that they would leave [A] |
| 31. I prefer just to let things happen rather than try to understand why they turned out that way [C] |
| 32. When I am confused by a problem, one of the first things I do is survey the situation and consider all the relevant pieces of information (r) [R]  **33. I don’t like to get involved in listening to another person’s troubles [A]**  34. I am hesitant about making important decisions after thinking about them [C]  35. Before criticizing somebody, I try to imagine how I would feel if I were in their place (r) [R]  36. I’m easily irritated by people who argue with me [A]  **37. When I look back on what’s happened to me, I feel cheated [R]**  38. Simply knowing the answer rather than understanding the reasons for the answer to a problem is fine with me [C] |
| 39. I sometimes find it difficult to see things from another person’s point of view [R] |

Response Scales: Strongly agree (1), Agree (2), Neutral (3), Disagree (4), Strongly disagree (5)

Short Version [New short Three-Dimensional Wisdom Scale (3D-WS)] items are in bolt. (r): reversed item.

Letters in Square brackets stand for the dimensions: [A] Affective, [C] Cognitive, [R] Reflective
